# Recurrence rate of perianal and intra-anal genital warts treated with aminolevulinic acid photodynamic therapy: a meta-analysis

**DOI:** 10.3389/frph.2025.1746426

**Published:** 2026-01-15

**Authors:** Mingqiang Liu, Fengming Hu, Guolan Li, Changxia Li, Hong Peng, Xiaohua Tao

**Affiliations:** 1 Dermatology, Dermatology Institute of Jiangxi Province, Nanchang, China; 2 Dermatology, Candidate Branch of National Clinical Research Center for Skin Diseases, Nanchang, China; 3 Dermatology, Jiangxi Provincial Clinical Research Center for Skin Diseases, Nanchang, China; 4 Dermatology, Dermatology Hospital of Jiangxi Province, Nanchang, China; 5 Dermatology, The Affiliated Dermatology Hospital of Nanchang University, Nanchang, China; 6 Dermatology, Jiangxi University of Traditional Chinese Medicine, Nanchang, China

**Keywords:** aminolevulinic acid, genital warts, meta-analysis, perianal and intra-anal, photodynamics

## Abstract

**Objective:**

This meta-analysis and systematic review aimed to evaluate the recurrence rate and clinical efficacy of aminolevulinic acid photodynamic therapy (ALA-PDT) in the treatment of perianal and intra-anal genital warts.

**Methods:**

We systematically searched the China National Knowledge Infrastructure (CNKI), Wanfang Data, VIP, Chinese Biomedicine (CBM), PubMed, and Cochrane Library databases from their inception to April 1, 2024, for randomized controlled trials (RCTs) investigating ALA-PDT for perianal and intra-anal genital warts. Study quality was assessed using the Cochrane Collaboration's risk of bias tool, and statistical analysis was performed using RevMan 5.3 software.

**Results:**

After rigorous screening, 32 RCTs involving 2,538 patients were included. The recurrence rate of perianal and intra-anal genital warts treated with ALA-PDT was significantly lower than that in conventional treatment groups (OR = 0.17, 95% CI [0.13, 0.22], *P* < 0.00001). While ALA-PDT monotherapy demonstrated lower efficacy compared to conventional treatments (OR = 0.49, 95% CI [0.29, 0.85], *P* = 0.01), ALA-PDT combined with other therapeutic modalities showed superior clinical outcomes (OR = 4.72, 95% CI [3.53, 6.32], *P* < 0.00001).

**Conclusion:**

ALA-PDT effectively reduces recurrence rates of perianal and intra-anal genital warts and enhances treatment efficacy when used in combination with conventional therapies. As all included studies were conducted in China, further multinational RCTs are warranted to validate these findings across diverse populations.

**Systematic Review Registration:**

https://www.crd.york.ac.uk/PROSPERO/, identifier CRD42024501535.

## 1 Introduction

Genital warts, caused by human papillomavirus (HPV), represent a prevalent sexually transmitted infection worldwide, with frequent occurrence in the perianal region, perineum, and genitals. The estimated global incidence ranges from 160 to 289 per 100,000 person-years (1), with prevalence rates of 103–168 per 100,000 in men and 76–191 per 100,000 in women (1). Large-scale epidemiological studies report recurrence rates of 47–163 per 100,000 in men and 23–110 per 100,000 in women (2–5). Due to the unique physiological structure of the perianal area and anal canal, which features many folds and rich local blood supply, treatment is difficult and the recurrence rate is high,perianal and intra-anal warts are particularly challenging to manage and are associated with higher recurrence rates following treatment, with intra-anal lesions being especially insidious.

Current therapeutic approaches for anogenital warts—including topical imiquimod, cryotherapy, CO₂ laser, and surgical excision—demonstrate moderate efficacy in clearing visible lesions but are limited by high recurrence rates. This often necessitates repeated treatment cycles, significantly increasing patient discomfort and economic burden.

Photodynamic therapy (PDT) represents an innovative treatment modality that combines photosensitizing agents with specific wavelength light exposure. The mechanism involves selective uptake of photosensitizers by diseased cells, followed by targeted irradiation that generates cytotoxic reactive oxygen species, thereby precisely destroying pathological tissue while preserving normal structures (6). Although ALA-PDT shows considerable promise in reducing recurrence of perianal and intra-anal warts, high-quality randomized controlled trials remain limited, and comprehensive systematic evaluations are lacking. This meta-analysis aims to synthesize existing evidence from RCTs to clarify the recurrence rate and therapeutic efficacy of PDT in managing perianal and intra-anal genital warts.

## 2 Materials and methods

### 2.1 Literature search strategy

Before conducting the research, we registered on the Prospero (https://www.crd.york.ac.uk/PROSPERO/) and carried out the study according to the relevant protocol steps.

We conducted a comprehensive search of CNKI, Wanfang, VIP, CBM, PubMed, and Cochrane Library databases using a combination of medical subject headings and free-text terms. Key search terms included: “anogenital warts,” “genital warts,” “anal warts,” “anal condyloma acuminata,” “aminolevulinic acid photochemotherapy,” and “aminolevulinic acid photodynamics.” The search period encompassed all available literature from database inception to April 1, 2024.

### 2.2 Inclusion criteria

Studies meeting the following criteria were included: (1) randomized or quasi-randomized controlled trials; (2) publications in Chinese or English; (3) participants with clinically or histopathologically confirmed genital warts located in perianal regions or anal canal; (4) no restrictions regarding age, sex, ethnicity, or race; (5) primary outcome including post-treatment recurrence rate; (6) experimental intervention consisting of ALA-PDT alone or combined with other therapies; and (7) outcome measures encompassing clinical efficacy and adverse reactions. Studies failing to meet these criteria were excluded.

### 2.3 Data extraction and quality assessment

Two investigators independently screened potential studies, extracted relevant data, and assessed methodological quality using the Cochrane Risk of Bias tool. Discrepancies were resolved through consensus discussion or arbitration by a third reviewer. All data were systematically managed using WPS Office Excel.

### 2.4 Data analysis

Statistical analyses were performed using RevMan 5.3 software. Continuous outcomes were expressed as weighted mean differences or standardized mean differences with 95% confidence intervals (CIs), while dichotomous outcomes were presented as odds ratios (ORs) with 95% CIs. Heterogeneity was quantified using the *I*^2^ statistic, with *I*^2^ > 50% indicating substantial heterogeneity warranting a random-effects model; otherwise, a fixed-effects model was applied. Sensitivity and descriptive analyses were conducted where significant clinical heterogeneity was present.

For the included quasi-randomized trials, a stepwise exclusion method can be used. Exclude one quasi-randomized trial at a time, then re-conduct the meta-analysis to observe the changes in the overall effect size. If the exclusion of any single quasi-randomized trial results in a change in the overall effect size of less than 10% and does not alter the statistical significance (*P*-value), it indicates that “the quasi-randomized trial has little impact on the overall results, and the results are robust.” If the exclusion of one or more quasi-randomized trials results in a change in the overall effect size of 10% or more, or if the statistical significance changes from “significant” to “not significant” (or vice versa), it indicates that “this quasi-randomized trial is a key study affecting the results,” and the baseline balance, interventions, outcome measurements, and other details of the study need to be individually checked to analyze the sources of bias. Publication bias was assessed through funnel plot symmetry analysis.

## 3 Results

### 3.1 Literature search results

**Figure 1:**
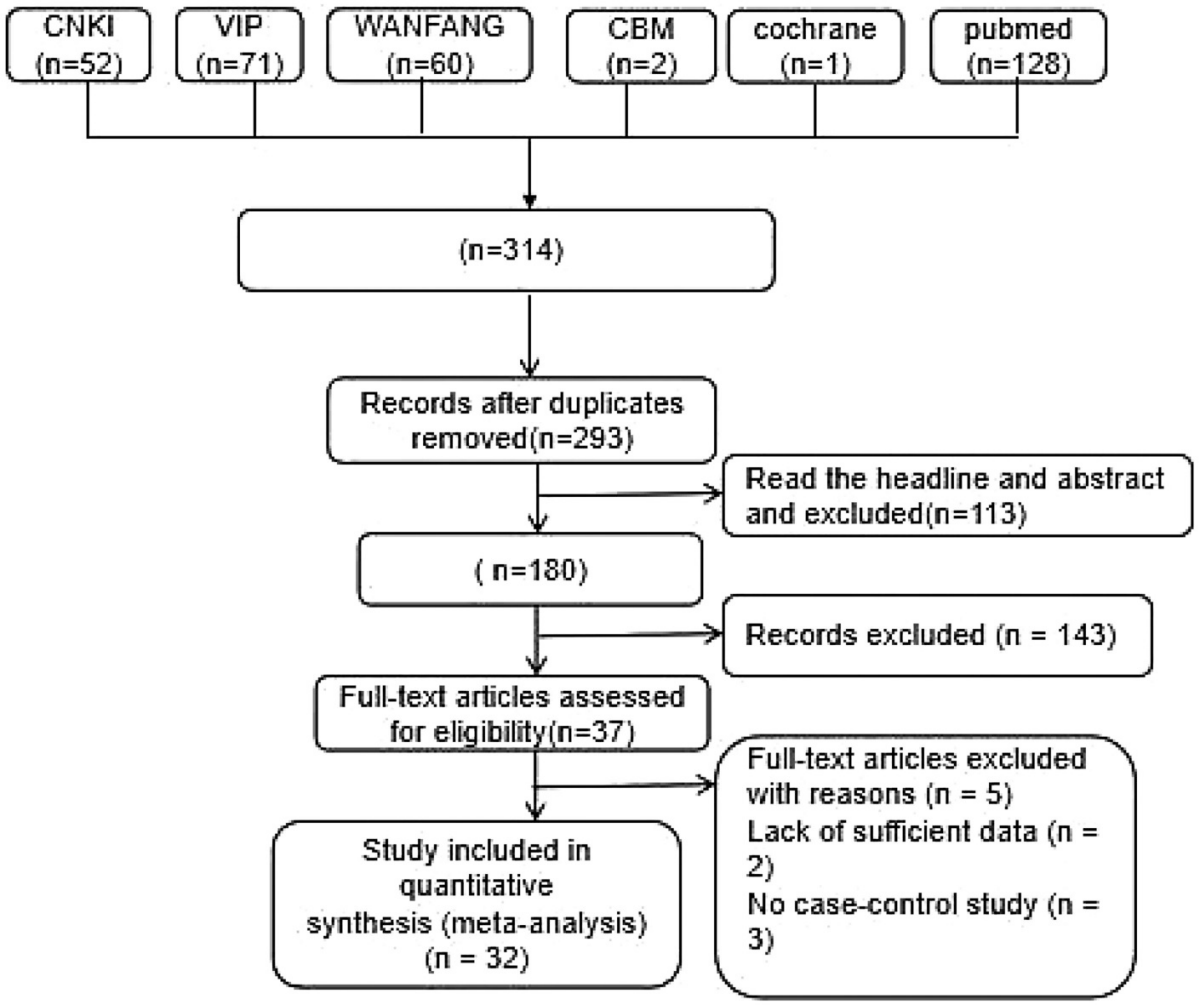
Flowchart of literature screening.

Our initial search identified 314 records. After duplicate removal, 293 unique articles underwent title and abstract screening, yielding 37 potentially eligible studies. Following full-text review, 32 RCTs (7–38) met inclusion criteria for meta-analysis. All included studies were conducted in China and published in Chinese. The study selection process is detailed in Figure 1, with study characteristics summarized in Tables 1, 2.

**Table 1:** Literature feature table.

FIRST author	Cases (T/C)	Treatment		Course	Gender (Male/Female)		Age (y)		Evaluation indicators
		T	C		T	C			
Yin (7)	40/35	High-frequency ionization therapy + ALA-PDT	High-frequency ionization therapy	3 w	27/13	24/11	17–65		①③
Yuan (9)	55/55	ALA-PDT	CO_2_ laser	24 w	Male		19–52	18–50	①③
Yang (11)	34/34	Liquid nitrogen cryotherapy + ALA-PDT	Liquid nitrogen cryotherapy	4 w	23/11	21/13	22–50	25–51	①②④
Zhang (12)	26/26	cryotherapy + ALA-PDT	Cryotherapy	30 d	37/15		18–56		①③
Zhang (14)	43/43	Microwave + ALA-PDT	Microwave	3 w	68/18		21–76		①②③
Zhang (15)	24/24	Microwave + ALA-PDT	Microwave	6 w	Male		*n*	*n*	①②③
Guo (16)	60/60	CO_2_ laser + ALA-PDT	CO_2_ laser	3 w	30/30	35/25	37.51 ± 0.52	37.56 ± 0.53	①②③
Zhu (17)	47/45	CO_2_ laser + ALA-PDT	CO_2_ laser	3 w	41/6	42/3	17–55	21–54	①②③
Zhu (18)	33/33	CO_2_ laser + Wart removal external lotion + ALA-PDT	CO_2_ laser + Wart removal external lotion	30 d	30/3	31/2	21–59	24–52	①②③
Wang (19)	28/28	CO_2_ laser + ALA-PDT	CO_2_ laser	20 d	25/3	26/2	18–56	20–60	①③
Huang (20)	31/31	ALA-PDT	CO_2_ laser、High-frequency electric knife	3 w	Male		18–46	20–47	①②③
Li (22)	40/40	CO_2_ laser + interferon gel + ALA-PDT	CO_2_ laser + interferon gel	3 m	23/17	22/18	18–53	19–52	①②③
Guo (23)	58/46	CO_2_ laser + ALA-PDT	CO_2_ laser	3 w	30/28	29/17	17–60	17–60	①②③
Li (24)	14/14	CO_2_ laser + ALA-PDT	CO_2_ laser	1 m	11/3	12/2	34.86 ± 11.26	35.65 ± 11.43	①②④
Liang (25)	72/66	CO_2_ laser + ALA-PDT	CO_2_ laser	3 w	126/18		18–58		①③
Wang (26)	43/43	CO_2_ laser + ALA-PDT	CO_2_ laser	6 w	25/18	24/19	19–59	19–61	①②③
Yi (27)	29/29	CO_2_ laser + ALA-PDT	CO_2_ laser	3 w	Male		17–47	19–46	①②③
Wang (28)	21/20	CO_2_ laser + ALA-PDT	CO_2_ laser	3 w	30/16		21–65		①②③
Guo (29)	45/45	Argon plasma coagulation + ALA-PDT	Argon plasma coagulation	3 w	69/21		17–52		①②③
Mi (31)	41/47	CO_2_ laser + ALA-PDT	CO_2_ laser	42 d	*N*	*N*	17–54	16–61	①②③
Su (32)	35/35	High-frequency ionization therapy + ALA-PDT	High-frequency ionization therapy	30 d	40/27		15–65		①②③
Liu (33)	60/30	surgery + ALA-PDT	Surgery	20 d	48/12	22/8	18–56	21–48	①②
Tang (34)	68/62	Argon high-frequency electric knife + ALA-PDT	Argon high-frequency electric knife	3 w	106/24		18–58		①③
Li (35)	43/43	Liquid nitrogen cryotherapy + ALA-PDT	Liquid nitrogen cryotherapy	4 w	Male		3,762 ± 79	3,716 ± 82	①②③
Zheng (36)	64/64	CO_2_ laser + ALA-PDT	CO_2_ laser	5w	86/42		21–56		①②

T, treatment group; C, control group; Evaluation indicators: ① Recurrence rate, ② Efficient, ③ Adverse effects, ④ HPV DNA load test.

**Table 2:** Literature feature table.

FIRST author	Cases (T/C1/C2)	Treatment			Course	Gender (Male/Female)			Age (y)	Evaluation indicators
		T	C1	C2		T	C1	C2		
Yin (8)	78/78/78	High-frequency electric knif + ALA-PDT	High-frequency electric knif	ALA-PDT	30 d	144/90			21–65	①②③
Hu (10)	30/30/30	High-frequency ionization therapy + ALA-PDT	High-frequency ionization therapy	ALA-PDT	3 w	19/11	19/11	17/13		①②③
Cai (13)	43/40/40	Liquid nitrogen cryotherap + ALA-PDT	Liquid nitrogen cryotherap	ALA-PDT	4 w	87/36			17–68	①②③
Zhu (21)	13/16/49	CO_2_ laser + ALA-PDT	CO_2_ laser	ALA-PDT	4 w	30/19	11/5	10/3	16–55	①③
Lin (30)	48/48/48	ALA-PDT+ High-frequency electric knife	High-frequency electric knife	ALA-PDT	3 w	25/23	26/22	27/21		①②③
Luo (37)	46/46/46	High-frequency ionization therapy + ALA-PDT	High-frequency ionization therapy	ALA-PDT	2 w	29/17	28/18	25/21	18–68	①②③
Wu (38)	22/23/22	Fulguration + ALA-PDT	Fulguration	ALA-PDT	4 m	12/10	14/9	14/8	36. 2/36. 4/34. 6	①②③

T, treatment group; C, control group; Evaluation indicators: ① Recurrence rate, ② Efficient, ③ Adverse effects.

### 3.2 Quality assessment of included studies

**Figure 2:**
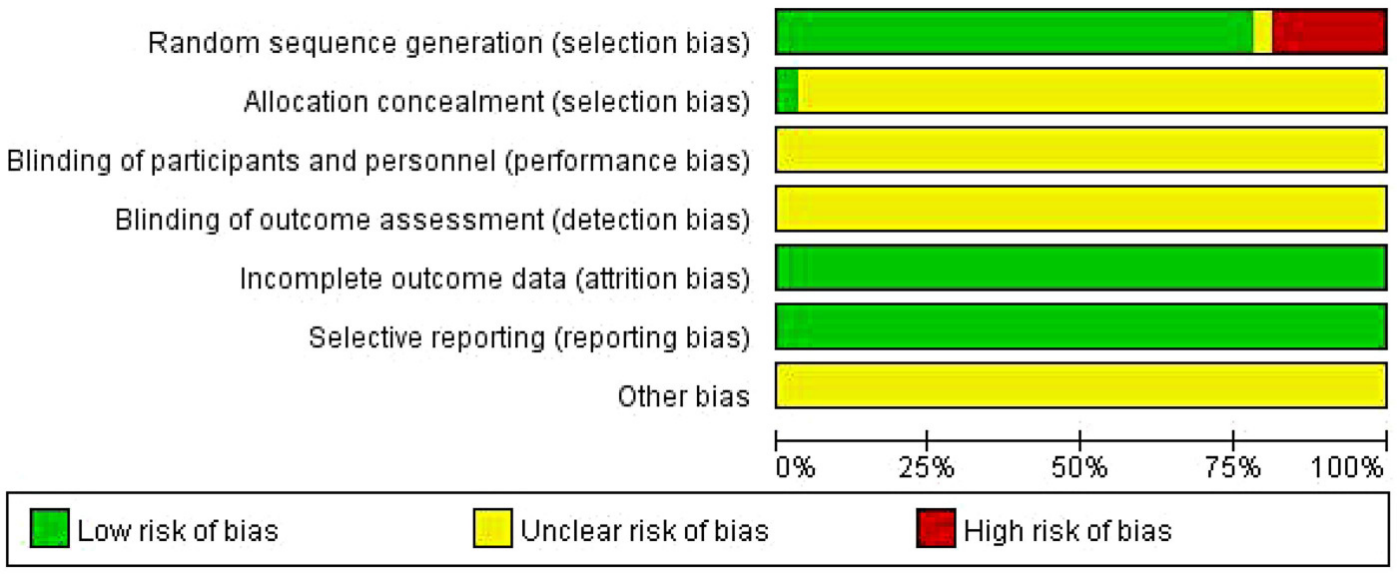
Analysis of literature quality and bias.

**Figure 3:**
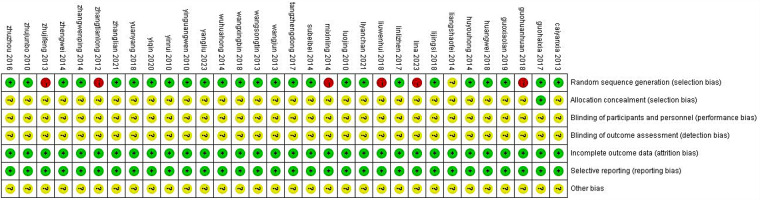
Summary of literature quality and bias analysis.

Among the 32 included studies, none reported blinding of participants or personnel, or allocation concealment. Three studies (14, 16, 33) did not employ proper randomization methods, while the remaining studies utilized various randomization approaches including consultation order (10, 13, 18, 20, 23, 25, 27, 30, 38), random grouping (15, 29), or random number tables (7, 11, 37). All studies demonstrated complete outcome data without evidence of selective reporting. Quality and bias assessments are presented in Figures 2, 3.

### 3.3 Recurrence rate analysis

#### 3.3.1 ALA-PDT combined with other therapies

**Figure 4:**
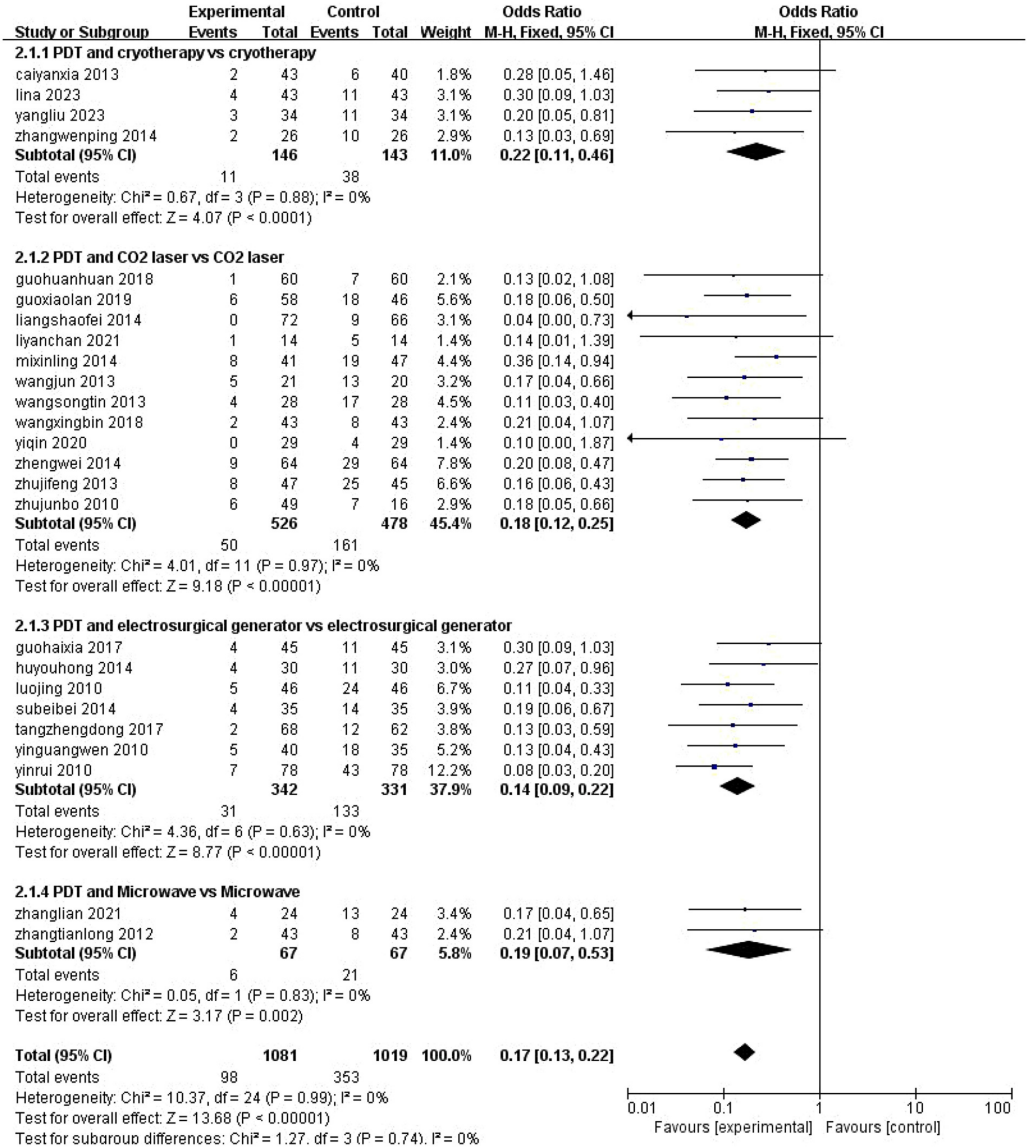
Meta-analysis of recurrence rates of ALA-PDT in combination with different treatment modalities. M-H, Mantel-Haenszel; CI, confidence interval.

Thirty-one studies (7, 9–38) reported recurrence rates for ALA-PDT combination therapy. Subgroup analyses demonstrated low heterogeneity, supporting use of a fixed-effects model. Specific findings included:Cryotherapy combination [4 studies (11–13, 35)]: OR = 0.22, 95% CI [0.11, 0.46], *P* < 0.0001; CO₂ laser combination [12 studies (16, 17, 19, 21, 23–28, 31, 36)]: OR = 0.18, 95% CI [0.12, 0.25], *P* < 0.00001; Electrosurgery combination [7 studies (7, 8, 10, 20, 30, 32, 34)]: OR = 0.14, 95% CI [0.09, 0.22], *P* < 0.00001;Microwave therapy combination (2 studies (14, 15): OR = 0.19, 95% CI [0.07, 0.53], *P* = 0.002;The pooled recurrence rate for combination therapy was OR = 0.17, 95% CI [0.13, 0.22], *P* < 0.00001 (Figure 4).

#### 3.3.2 ALA-PDT monotherapy

**Figure 5:**
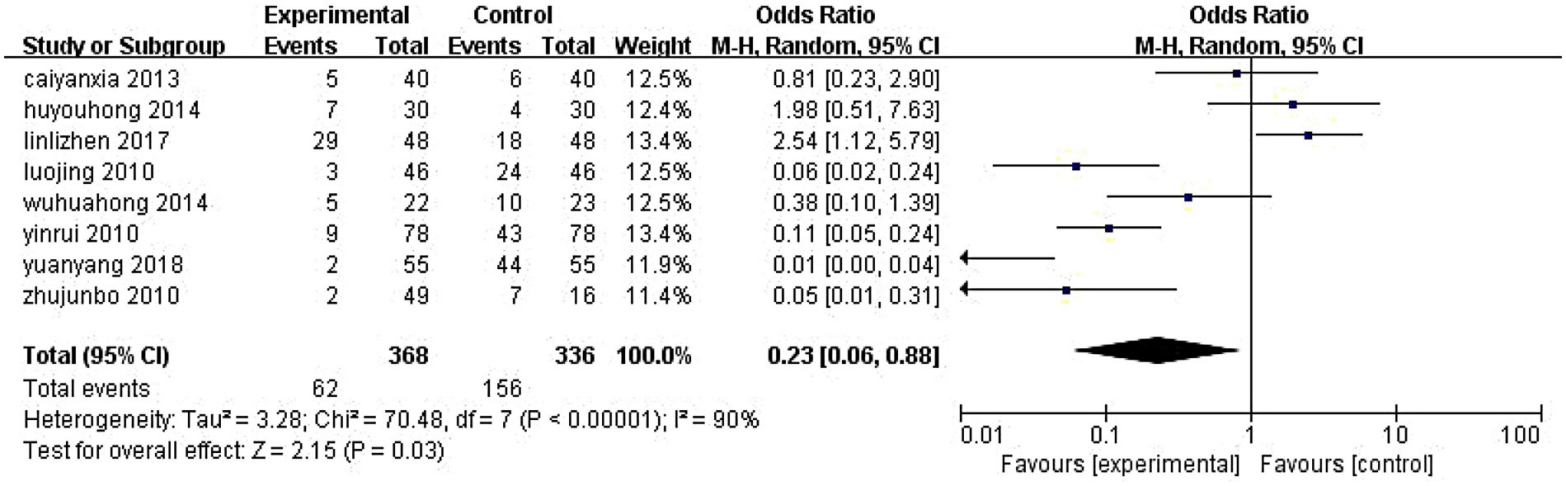
Meta-analysis of recurrence rates with ALA-PDT alone. M–H, Mantel–Haenszel; CI, confidence interval.

Eight studies (8–10, 13, 21, 30, 37, 38) evaluated ALA-PDT monotherapy. Significant heterogeneity was observed (*P* < 0.00001, *I*^2^ = 90%), necessitating a random-effects model. The recurrence rate was OR = 0.23, 95% CI [0.06, 0.88], *P* = 0.03 (Figure 5).

### 3.4 Efficacy analysis

#### 3.4.1 ALA-PDT combination therapy

**Figure 6:**
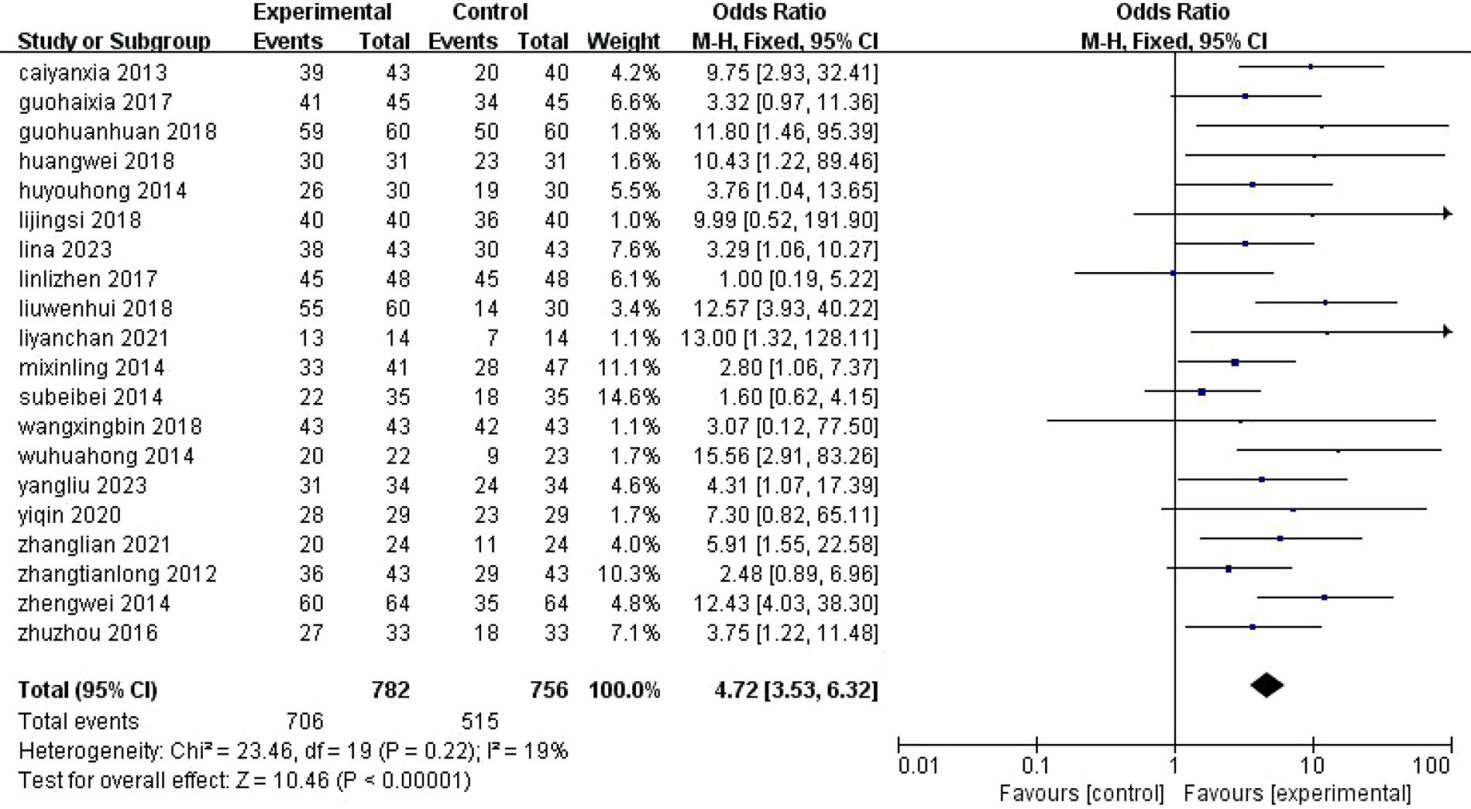
Meta-analysis of the effectiveness of ALA-PDT in combination with different treatment modalities. M–H, Mantel–Haenszel; CI, confidence interval.

Twenty studies (10–16, 18, 20, 22, 24, 26, 30–33, 35) reported efficacy outcomes for combination therapy. Heterogeneity was low (*I*^2^ = 19%), and fixed-effects model analysis demonstrated significantly superior efficacy for combination therapy vs. control (OR = 4.72, 95% CI [3.53, 6.32], *P* < 0.00001) (Figure 6).

#### 3.4.2 ALA-PDT monotherapy

**Figure 7:**
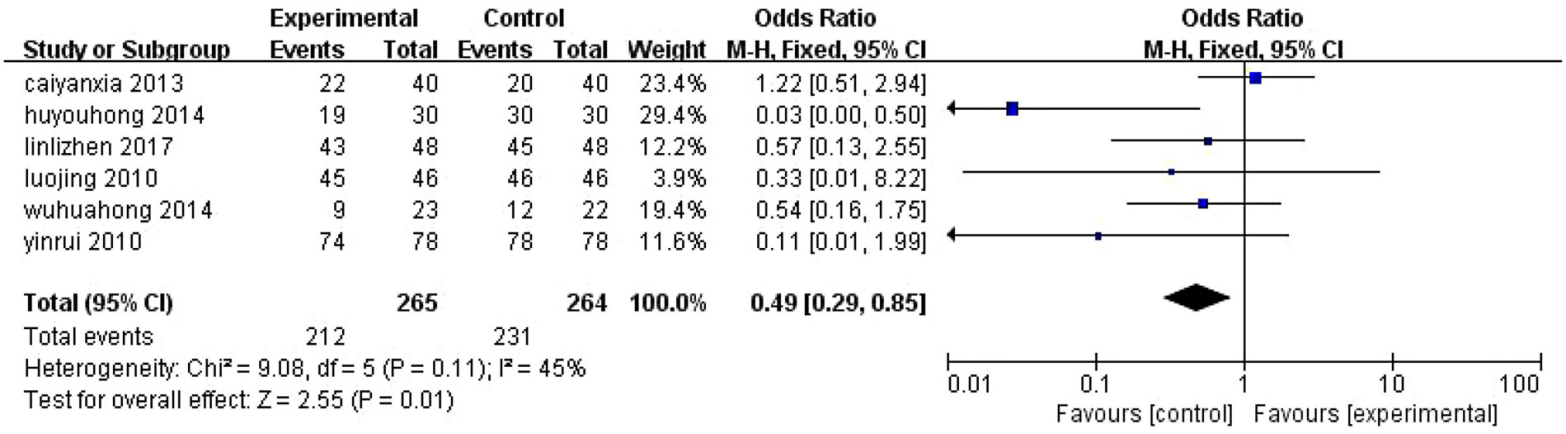
Meta-analysis of the effectiveness of ALA-PDT alone. M–H, Mantel–Haenszel; CI, confidence interval.

Six studies (8, 10, 13, 30, 37, 38) reported monotherapy efficacy. Moderate heterogeneity was observed (*I*^2^ = 45%), and fixed-effects model analysis revealed lower efficacy for ALA-PDT alone vs. control (OR = 0.49, 95% CI [0.29, 0.85], *P* = 0.01) (Figure 7).

### 3.5 Publication bias

**Figure 8:**
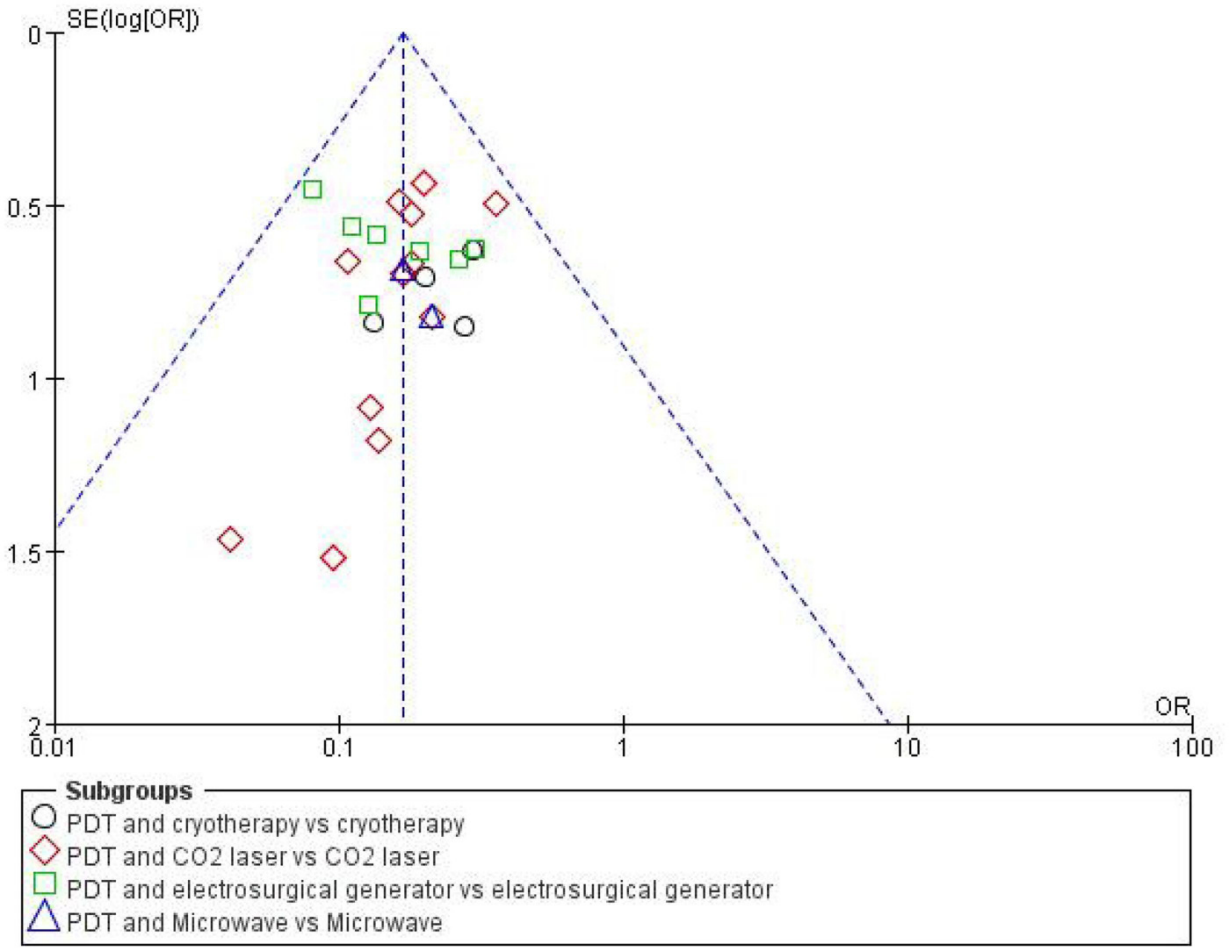
Funnel plot for bias analysis.

Funnel plot analysis of the 31 included studies demonstrated general symmetry, though two CO_2_ laser studies exhibited slight bias, suggesting acceptable overall publication bias (Figure 8).

## 4 Discussion

### 4.1 Pathogenesis and treatment challenges

Perianal and intra-anal condyloma acuminatum are predominantly caused by low-risk HPV types 6 and 11 (41), with HPV 11 demonstrating particular association with elevated recurrence risk (42). The anatomical vulnerability of anal canal mucosa to microtrauma during sexual intercourse, combined with the warm, moist local environment, creates ideal conditions for HPV persistence and replication (1, 43). Without appropriate intervention, these lesions may progress to giant condylomata (Buschke-Löwenstein Tumors), characterized by deep tissue infiltration and fistula formation, presenting substantial therapeutic challenges (44). In severe cases, untreated BLT has been associated with fatal outcomes due to secondary complications (44).

Conventional therapies including laser ablation, cryotherapy, topical agents, and surgical excision effectively remove visible wart tissue but fail to eradicate subclinical and latent HPV infections in surrounding normal-appearing skin (45, 47). The reactivation of these residual viral reservoirs constitutes the primary mechanism underlying the high recurrence rates observed with conventional treatments, creating a cycle of recurrence and retreatment that significantly impacts patients' quality of life and economic burden.

### 4.2 Mechanisms and advantages of ALA-PDT

Photodynamic Therapy represents a targeted treatment modality with established efficacy across various conditions, including neoplastic, inflammatory, and infectious diseases (46, 55). The mechanism involves topical application of 5-aminolevulinic acid, which is selectively metabolized to protoporphyrin IX in HPV-infected cells. Subsequent irradiation with red light at 630 nm in wavelength activates PpIX, generating cytotoxic reactive oxygen species that induce selective apoptosis and necrosis of target cells while minimizing damage to healthy tissue (53, 54, 56).

In the context of perianal and intra-anal CA, PDT offers several distinct advantages:

Comprehensive Infection Clearance: PDT enables treatment of entire anatomical fields, effectively targeting and eliminating subclinical and latent HPV infections that constitute primary sources of recurrence (47, 48, 56).

Immune Activation: Beyond direct cytotoxic effects, PDT stimulates localized immune responses by enhancing dendritic cell infiltration, improving CD4+ T-cell function, promoting interferon secretion, and modulating immune-related markers including TLR4 and NF-κB. This helps reverse HPV-induced local immunosuppression, contributing to sustained clearance (50–52).

Tissue Preservation: The selective nature of PDT makes it particularly suitable for anatomically complex regions like the anal canal, where preserving mucosal integrity and sphincter function is paramount. This approach minimizes risks of stenosis, scarring, and functional impairment associated with more destructive modalities (49).

Synergistic Potential: Combination with ablative methods like CO_2_ laser addresses PDT's penetration limitations. Initial laser debulking of visible warts enhances ALA penetration, while subsequent PDT clears residual subclinical disease, creating synergistic effects for comprehensive lesion control (39, 40).

### 4.3 Clinical implications and evidence synthesis

Our meta-analysis confirms that ALA-PDT, particularly as an adjuvant to conventional therapies, significantly reduces recurrence rates in perianal and intra-anal CA. While monotherapy efficacy for bulky warts may not surpass conventional treatments, ALA-PDT's role in enhancing overall treatment success and preventing recurrence is well-established. The strategic approach of initial visible wart removal followed by ALA-PDT sessions to address subclinical infection represents an optimal clinical protocol (39).

### 4.4 Limitations and future directions

This study has several limitations. The exclusive inclusion of Chinese studies restricts generalizability to other populations. Treatment-associated pain may impact patient compliance, and methodological challenges in blinding affect trial quality. I believe that in the precise management of pain, mild pain can be treated with local anesthetics, moderate pain can be managed with local nerve blocks or nerve tissue interventions, and severe pain can be treated with general anesthesia. This approach will allow for better pain management and increase patient compliance with the treatment (57). Additionally, the absence of subgroup analyses based on age or sex limits demographic-specific insights.

Future research should focus on:.

Developing novel photosensitizers with enhanced tissue penetration and improved tolerability.

Optimizing light delivery parameters and exploring fractionated irradiation protocols.

Investigating combination strategies with immunomodulators and therapeutic HPV vaccines.

Establishing standardized treatment protocols to facilitate clinical implementation.

## 5 Conclusion

Although this method was included in quasi-randomized trials and carries a relatively high risk of bias, sensitivity analysis indicates that this bias has little impact on the overall conclusion, which remains robust. The tendency of anal condylomas to recur imposes a significant financial and psychological burden on patients, so addressing the recurrence of this disease can significantly reduce patient burden (58). When ALA-PDT is used in combination with conventional therapies (such as carbon dioxide laser), it can significantly reduce recurrence rates and improve the treatment efficacy of perianal and intra-anal genital warts. Its targeted mechanism, tissue-preserving characteristics, and immune-modulating effects make it a valuable minimally invasive option, particularly suitable for complex, recurrent, or extensive cases. Future multinational studies and protocol standardization will further solidify its place in clinical practice.

## Data Availability

The original contributions presented in the study are included in the article/Supplementary Material, further inquiries can be directed to the corresponding authors.
